# Assessment of gastrointestinal function and enteric nervous system changes over time in the A53T mouse model of Parkinson’s disease

**DOI:** 10.1186/s40478-025-01956-7

**Published:** 2025-03-12

**Authors:** Myat Noe Han, Madeleine R. Di Natale, Enie Lei, John B. Furness, David I. Finkelstein, Marlene M. Hao, Shanti Diwakarla, Rachel M. McQuade

**Affiliations:** 1 Department of Anatomy and Physiology, University of Melbourne, Parkville VIC, Melbourne, 3010 Australia; 2 The Florey Institute of Neuroscience and Mental Health, University of Melbourne, Parkville, VIC 3010 Australia; 3 Gut Barrier and Disease Laboratory, Department of Anatomy and Physiology, University of Melbourne, Parkville VIC, Melbourne, 3010 Australia

**Keywords:** Parkinson’s disease, Gastrointestinal dysfunction, Constipation, A53T, Enteric nervous system

## Abstract

Gastrointestinal (GI) dysfunctions, including constipation and delayed stomach emptying, are prevalent and debilitating non-motor symptoms of Parkinson’s disease (PD). These symptoms have been associated with damage in the enteric nervous system (ENS) and the accumulation of pathogenic alpha-synuclein (α-Syn) within the GI tract. While motor deficits and dopaminergic neuron loss in the central nervous system (CNS) of the A53T mouse model are well-characterised, the temporal relationship between GI dysfunction, ENS pathology, and motor symptoms remains unclear. This study aimed to investigate functional alterations in the GI tract at the early stages of the disease, before the appearance of motor deficits, both in vivo and ex vivo. Early colonic motility deficits observed in A53T mice, measured via bead expulsion, preceded motor impairments emerged at 36 weeks. Although whole-gut transit remained unchanged, reduced faecal output was concurrent with marked colonic dysmotility at 36 weeks. Despite a lack of significant neuronal loss, a greater number of enteric neurons in A53T mice showed signs of neuronal hypertrophy and increased nuclear translocation of HuC/D proteins indicative of neuronal stress at 12 and 36 weeks. Calcium imaging revealed differential enteric neuron activity, characterised by exaggerated calcium transients at 12 weeks that normalized by 36 weeks. Furthermore, a reduction in enteric glial populations was observed as early as 12 weeks in both the ileum and colon of A53T mice. These findings provide compelling evidence that ENS pathology, including neuronal stress, disrupted calcium signalling, and glial cell loss, precedes the onset of motor symptoms and may contribute to early GI dysfunction in PD.

## Introduction

Parkinson’s disease (PD) is a complex neurodegenerative disorder characterised by the loss of dopaminergic neurons in the substantia nigra pars compacta (SNpc) and the accumulation of pathogenic alpha-synuclein (α-Syn) aggregates within cytoplasmic inclusions termed Lewy bodies (LBs). The loss of dopaminergic neurons in the nigrostriatal pathway, and subsequent reduction of dopamine levels in the dorsolateral putamen [22], are responsible for the characteristic motor symptoms of PD, which include bradykinesia, resting tremor, rigidity, stooped posture, and loss of postural reflexes [13, 36]. However, in addition to the classic motor symptoms, PD is also associated with non-motor symptoms, reflecting the multi-system nature of the disease. Non-motor symptoms include gastrointestinal (GI) and olfactory deficits, as well as neuropsychiatric and sleep disorders, all of which negatively affect patient quality of life [20].

Gastroparesis and constipation are among the most prevalent GI symptoms that affect PD patients and often manifest decades before clinical diagnosis [15, 46, 56, 93]. Despite attempts to understand the pathophysiological underpinnings of these symptoms, the causes of PD-related GI deficits remain unclear [38, 56]. However, the identification of pathological α-Syn-enriched aggregates throughout the GI tract and evidence of neuropathy in the nervous system of the gut in both animal models of PD and in human patients suggests pathogenic forms of α-Syn may drive PD-related GI symptoms [12, 53, 89, 92, 94, 95, 108].

The GI system is regulated by the enteric nervous system (ENS), a part of the autonomic nervous system, along with its connections to the central nervous system (CNS) [39]. The ENS is embedded in the gut wall and organised into two ganglionated networks in most regions. The submucosal plexus is found within the connective tissues of the submucosa, and the myenteric plexus lies between the circular and longitudinal layers of smooth muscle and runs the entire length of the GI tract [41]. These enteric neurons interact with the CNS to regulate contraction, relaxation, secretion and absorption of nutrients [40]. Several PD clinical studies have identified LBs and/or the presence of enteric neuropathy in the myenteric plexus and submucosal plexus of the colon alongside the manifestation of GI symptoms [47, 58–60, 85, 96, 106, 107]. This link between pathogenic α-Syn and GI dysfunction is further supported by animal models of PD, where pathogenic α-Syn fibrils injected into the lining of the GI tract are hypothesised to cause GI deficits and physiological changes to the ENS [19].

Notably, sex differences have emerged as an important factor in both PD pathology and GI dysfunction. Epidemiological studies suggest that males have a higher risk of developing PD, yet females more frequently report severe non-motor symptoms, including GI dysfunction [69, 111]. These differences may be influenced by sex hormones, immune responses, and variations in ENS structure and function. In this study, we assessed the role of the ENS on GI dysfunction in PD using the A53T mouse model, which overexpresses the human mutation of the α-Syn gene associated with familial forms of PD. This mutation leads to an increased propensity of α-Syn protein to misfold, aggregate, and form toxic species, closely mimicking the pathology observed in the human condition [43]. A53T mice exhibit key features of human PD, including progressive loss of dopaminergic neurons in the substantia nigra, α-Syn aggregates (Lewy bodies and Lewy neurites) in the CNS, motor deficits, and cognitive impairments; importantly, this model has been extensively studied [112]. Although some studies have reported GI symptoms in A53T mice [31, 32, 57, 89, 105, 109], few studies have investigated the timing of GI symptom onset and the associated cellular changes within the ENS. Therefore, this study aimed to determine whether the onset of GI symptoms correlated with alterations in neuronal and glial populations in the ENS.

## Methods

### Ethics approval

All procedures involving mice were performed in accordance with “Principles of laboratory animal care” (NIH publication No. 86 − 23, revised 1985) and the guidelines of the National Health and Medical Research Council (NHMRC), Australian code of practice for the care and use of animals for scientific purposes. All experiments were approved by the Florey Institute for Neuroscience and Mental Health Animal Ethics Committee (AEC No. 18–113) and comply with the ARRIVE guidelines.

### Animals

Mice were group housed (2–5 animals/cage) in a temperature- and humidity-controlled room under a 12-hour light/dark cycle. Food and water were available *ad libitum*. Mice (B6; C3-Tg-Prnp/SNCA*A53T/83Vle/J) were originally obtained in breeding pairs from Jackson Laboratories (Bar Harbor, ME). A breeding colony of transgenic mice that carry the human A53T mutation, driven by the mouse prion promoter, was established from A53T heterozygous breeders to produce both wild-type (WT) and homozygous transgenic (A53T) mice [43]. All mice used in this study were confirmed to have the transgene by qPCR. Separate cohorts of mice were humanely killed every 4 weeks (from 12 to 36 weeks of age). Cohorts contained an equal mixture of female and male mice. Sex-specific differences were assessed for all parameters via two-way ANOVA with Fisher’s LSD. Results were pooled due to no significant gender differences. A total of 160 mice were used in this study.

### Ledged beam test

Motor coordination and balance in WT and A53T mice were assessed using the ledged beam test, as previously described [31]. Briefly, a 1.5 m long beam, consisting of six 10 cm sections that progressively narrowed from 3.5 cm (Sect. 1) to 0.5 cm (Sect. 6) in 1 cm increments with a safety ledge on each side was utilised. The mice were trained to traverse the beam from the widest to the narrowest section, finishing in their home cage. Each mouse underwent two days of training, followed by testing on the third day. During the testing phase, the mice were recorded while traversing the beam in four trials with a 15- to 30-second rest period. The videos were analysed in slow motion by an investigator blinded to the genotype of the mice. The total number of foot faults (i.e. foot slipping onto the ledge) per section was averaged over the four trials, and the time taken to cross the beam was recorded to the nearest second.

### Faecal pellet output and pellet water content

Faecal pellet output (FPO), including number of pellets, pellet dry weight and pellet wet weight, was used as a measure of GI function. Mice were individually housed in a new cage, without bedding, food or water, for 30 min. Faecal pellets were collected immediately upon expulsion and placed into pre-weighed tubes. Water content was determined by drying the pellets overnight at 65 °C and reweighing the tubes. The percentage pellet water content was then calculated using the following formula: *[(stool wet weight − stool dry weight) / (stool wet weight)] × 100* [31].

### Bead expulsion test

Distal colonic transit time was evaluated using the bead expulsion test, which measures the duration required for mice to expel a bead inserted into the distal colon, as previously described [31]. Briefly, mice were lightly anesthetised with isoflurane (2–3%) to facilitate the insertion of a 3 mm bead approximately 2 cm into the distal colon. To minimise tissue damage, a flexible plastic rod was employed for bead insertion (Diwakarla et al., 2019). Post-insertion, the mice were placed in individual cages to recover from anaesthesia. The time from bead insertion to expulsion was recorded to the nearest second.

### Whole gut transit time test

Whole gut transit time (WGTT) was assessed following oral gavage with a 50% (v/v) cochineal extract solution prepared in drinking water (Queen Fine Foods, Alderley, QLD), as previously described (Diwakarla et al., 2019). After oral gavage, mice were individually housed in fresh cages with bedding and free access to food and water. Mice were monitored for up to 9 h, and the time to excretion of the first red pellet was recorded for each mouse. Mice that did not pass a red pellet within 9 h were scored as having a transit time of 9 h.

### Tissue collection

Animals were deeply anesthetized with isoflurane (inhalation) and perfused through the heart with 50 mL of ice-cold phosphate-buffered saline (PBS: 137 mM NaCl, 2.7 mM KCl, 10 mM Na_2_HPO_4_, and 1.8 mM KH_2_PO_4_, pH 7.2). The brain was dissected and perfused with 4% paraformaldehyde overnight and then placed in 30% sucrose in PBS at 4 °C for about a week. Duplicates of distal ileum and colon segments (3–5 cm) were dissected from the abdomen of each mouse and placed in ice-cold PBS. For wholemount preparations, the tissue was cut along the mesenteric border, cleared of contents, maximally stretched, and pinned mucosa side down. The tissues were then fixed with Zamboni’s fixative (2% formaldehyde, 0.2% picric acid in PBS) overnight at 4 °C. The fixed tissues were cleared of fixative with three 10-minute washes in dimethyl sulfoxide (DMSO) (Sigma-Aldrich, Australia) followed by three 10-minute washes in PBS. The tissues were stored at 4 °C in PBS containing 0.1% (w/v) sodium azide. For cross-sectional preparations, following dehydration of the tissue by incubation with 70% ethanol, it was paraffin-embedded, cut (5 μm thick sections) with a microtome, and stained for Hematoxylin and Eosin (H + E), Alcian blue, or used for immunohistochemistry. Tissue processing and histology staining was performed by the Melbourne Histology Platform (MHP).

### Stereology

Brains were sectioned and stained with neutral red to assess for total nigral neurons in the SNpc using stereology, as previously described [78]. Briefly, a complete series of 30 μm thick sections were cut and stained with neutral red (Nissl, Grale Scientific, Victoria, Australia) as previously described [2]. To estimate the total number of neurons in the SNpc, a fractionator sampling design was used [54, 63, 81]. Counts were taken at regular, predetermined intervals (x = 140 μm, y = 140 μm), with systematic area samples starting from a random point. An unbiased counting frame (45 μm x 35 μm) was superimposed over the tissue images using stereology software (MBF, Stereo Investigator) with a 63x objective lens (Leica, N.A.1.36). Experimenters were blinded to the genotype of each mouse.

### Immunohistochemistry and histology

For tissue preparation, myenteric plexus adhering to the longitudinal muscle layer were isolated by removing both the mucosa and circular muscle layers for wholemounts. For immunohistochemistry, the tissues were first blocked for 1 h at room temperature with 10% (v/v) normal horse serum (Sigma-Aldrich, Sydney, NSW, Australia) in PBS containing 1% Triton-X-100. Tissue was then incubated overnight at 4 °C with combinations of the following primary antibodies: human anti-HuC/D (1:20000; a gift from Dr Vanda Lennon, Mayo Clinic, USA), goat anti-neuronal nitric oxide synthase (nNOS; 1:1000, Abcam Cat#ab1376), goat anti-Sox10 (1:1000, R&D Systems Cat#AF2864), rabbit anti-S100β (1:1000, Abcam Cat#ab52642), goat anti-PGP9.5 (1:1000, ThermoFisher Cat#PA5-19349) or rabbit anti-phospho α-Syn (1:1000, Abcam Cat#ab51253). The wholemount and cross-section preparations were washed three times for 10 min each in PBS, followed by incubation with appropriate secondary antibodies for 2 h at room temperature. The secondary antibodies used were donkey anti-human Alexa 594 (1:500, Abacus Cat#709-585-149), donkey anti-goat 488 (1:500, ThermoFisher Scientific Cat#a32814), donkey anti-sheep 647 (1:500, ThermoFisher Scientific Cat#a21448), or donkey anti-rabbit 488 secondary antibodies (1:500, ThermoFisher Scientific Cat#a32790). Following this, tissue preparations underwent three 10-minute washes in distilled water and incubation with Hoechst 33,258 solution (10 µg/ml Bisbenzimide-Blue in distilled water; Sigma-Aldrich, Sydney, NSW, Australia) for 5 min. The tissues were given three additional washes in distilled water before being mounted on glass slides using a fluorescent mounting medium (Dako, Carpinteria, CA, USA).

### Calcium imaging

Live calcium (Ca^2+^) imaging was conducted on ex vivo preparations of circular muscle myenteric plexus (CMMP) from the colon. The CMMP preparation was mounted over a small inox ring as described previously [65]. CMMP preparations of the colon were loaded with Fluo4AM dye (Invitrogen/Molecular Probes Cat #F14217-500ul; 1 µM for 20 min), which fluoresces upon binding to calcium. All dissections and recordings were performed in Krebs’ solution (composition in mM: 117 NaCl, 4.6 KCl, 2.5 CaCl_2_, 1.2 MgSO_4_, 1 NaH_2_PO_4_, 25 NaHCO_3_, 11 D-glucose). Two solutions were used to induce a neuronal response: a high K^+^ Krebs solution, which artificially increases extracellular potassium concentrations (composition in mM: 73 NaCl, 50 KCl, 2.5 CaCl_2_, 1.2 MgSO_4_, 1 NaH_2_PO_4_, 25 NaHCO_3_, 11 D-glucose), and the nicotinic receptor agonist 1,1-dimethyl-4-phenylpiperazinium (DMPP, 10 µM), which induces depolarisation by opening ligand-gated ion channels (nicotinic receptors) upon binding. Imaging was performed using an inverted microscope (Axiovert 25, Zeiss, Jena, Germany) equipped with a ×20 (NA 0.5) objective lens and an Axiocam 702 mono camera (Zeiss, Jena, Germany). Videos, each 1 min in duration, were acquired at a rate of 2 Hz (image size 512 × 512 pixels). Cells were illuminated with a 470 nm LED (Zeiss Colibri) with an exposure time of 30–50ms per image. All recordings were conducted at room temperature.

Ca^2+^ imaging videos were analysed using Igor Pro software (Wavemetrics, Lake Oswego, OR, USA) with custom-written workflow [65]. A region of interest (ROI) was drawn over the cell body of each responding neuron to calculate the change in intracellular Ca^2+^ levels. Changes in fluorescence intensity were detected and expressed as fraction of the baseline fluorescence (F/F_0_), using the first 10 frames. The amplitude of Ca^2+^ transients were calculated from the baseline to the maximum fluorescence at the peak (∆F/F_0_) and compared between cells across different cohorts.

### Imaging and quantitative analysis

All images were captured using a ZEISS AXIOSCAN 7 (Carl Zeiss, Sydney, NSW, Australia) with a 10× air objective or an AxioImager.Z1 microscope with a 10× air objective (Carl Zeiss, Sydney, NSW, Australia). Quantification was performed using the Gut Analysis Toolbox (GAT) [98] in QuPath software [8]. The average number of Hu-positive neurons per area and the proportion of nNOS-positive neurons were quantified in a minimum of six randomly chosen regions (each 800 μm²) per preparation, encompassing approximately 200–400 cells per image. The same method was used to quantify the number of Sox-10-positive and S100β-positive glial cells.

The area of Hu-positive and nNOS-positive neurons was measured using the QuPath cell analysis function. Additionally, the ratio of Hu^+^ translocation was quantified in approximately 100–200 cells per preparation from images captured with a 20× air objective. The nucleus and cytosol of each neuron were traced separately using the freehand tool and the mean grey value of each was measured. Data is presented as a ratio of nuclear Hu: cytoplasmic Hu.

### Statistical analysis

Data are expressed as the mean ± standard deviation (SD). Comparisons between groups were performed using two-way ANOVA with the Uncorrected Fisher’s LSD test for multiple comparisons or one-way ANOVA followed by unpaired t-tests with Welch’s correction for multiple comparisons. Due to intragroup variability, bead expulsion and WGTT data were transformed into a natural log to normalize their distribution, stabilise variance, and reduce the impact of outliers, thereby enhancing the accuracy and interpretability of subsequent analyses. Data were analysed by multiple Kolmogorov-Smirnov (KS) tests for count data (ledged beam test and FPO) and two-way ANOVA with Fisher’s LSD test or multiple unpaired Welch’s t-test for other genotype comparisons. Prior to pooling, sex-differences were analysed by two-way ANOVA with Fisher’s LSD. Analyses were performed using GraphPad Prism (GraphPad Software Inc., San Diego, CA, USA). P values of less than 0.05 were considered statistically significant.

## Results

### A53T mice developed progressive central deficits when compared to WT mice

**Fig. 1:**
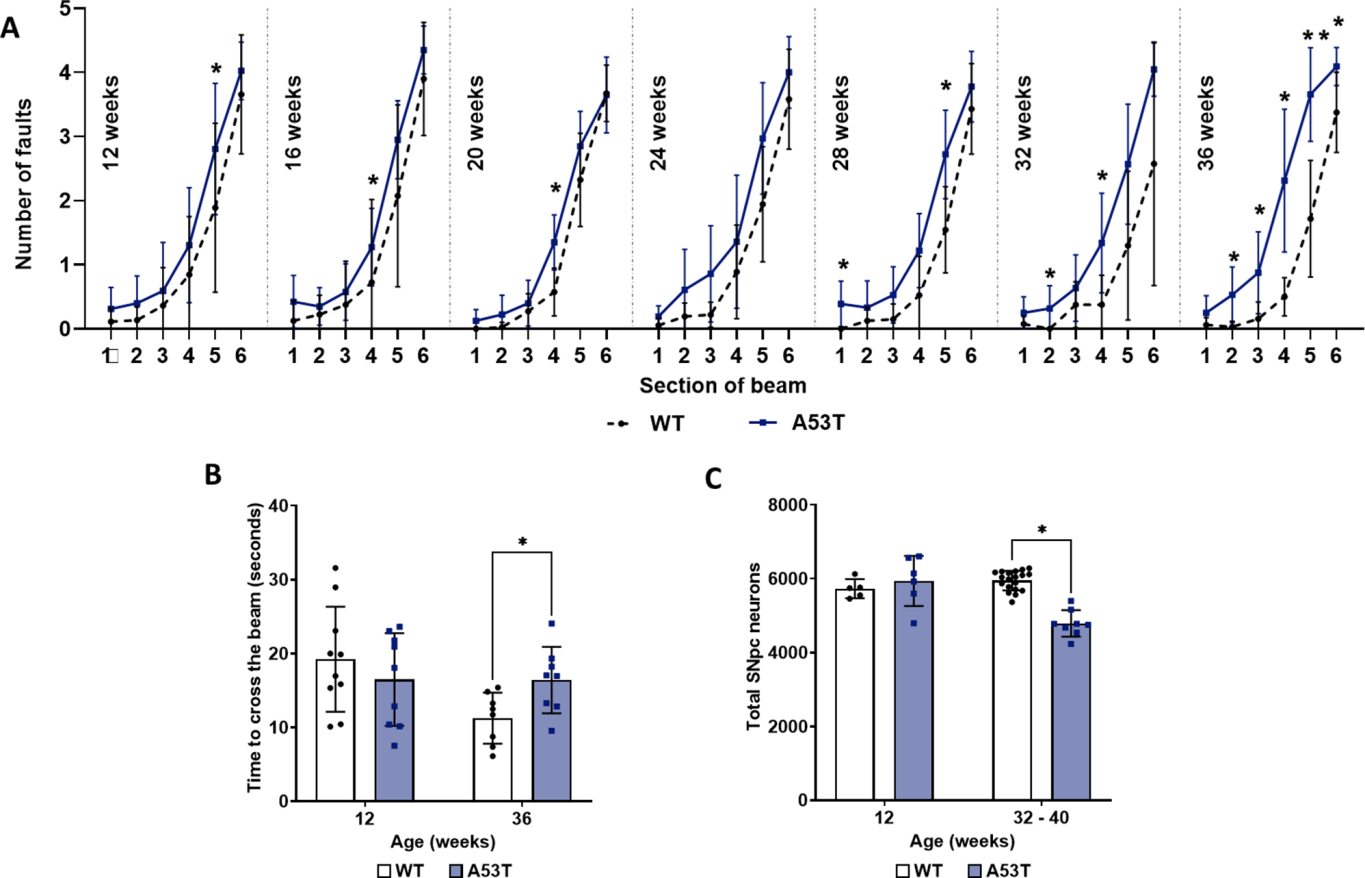
Assessment of central deficits in WT and A53T mice. (**A**) A53T mice exhibited a progressive deficit in motor function compared to WT mice, producing a significantly greater number of foot faults by 36 weeks of age. (**B**) Motor dysfunction was also evident in the time taken to cross the beam, with A53T mice taking significantly longer than WT mice at 36 weeks of age. (**C**) The total number of neurons in the SNpc was lower in A53T mice at 32–40 weeks. Data were analysed by multiple KS tests for genotype comparisons of foot fault count data (A; *n* = 8–20 per group) and using multiple unpaired Welch’s t-test for traversal time and cell number data (B; *n* = 8–10 per group, C; *n* = 5–19 per group). Data are expressed as mean ± SD. *p < 0.05, **p < 0.01

The ledged beam test was used to assess motor function—specifically coordination and balance—in A53T and WT mice. Results showed an age-related decline in motor coordination in A53T mice (Fig. 1A and B) with gradual progression of the deficit observed at 24, 28 and 32 weeks (Fig. 1A). By 36 weeks of age, A53T mice exhibited a significant increase in foot faults across nearly all sections of the beam, except the widest section (Sect. 1). Notably, foot faults were significantly higher in A53T mice between Sects. 2–4 and 5–6 (*p* < 0.01 and *p* < 0.05, respectively) when compared to WT mice. Additionally, traversal time across the beam was measured at 12 and 36 weeks; at 36 weeks, A53T mice took significantly longer than WT mice to cross the beam (*p* < 0.05; Fig. 1B), while traversal times were similar at 12 weeks. To enhance our analysis, we included an additional cohort of WT and A53T mice, performing stereological counts of nigral neurons in young mice (~ 12 weeks of age) and at a later stage when motor symptoms typically appear (~ 32 to 40 weeks) (Fig. 1C). A statistically significant decrease in nigral neuron count was seen at 32–40 weeks, supporting the presence of central deficits. This onset of motor dysfunction at 36 weeks aligns with previous findings [31, 77], identifying it as a key timepoint for the emergence of significant motor symptoms in this model.

### A53T mice exhibit early changes in colonic motility

**Fig. 2:**
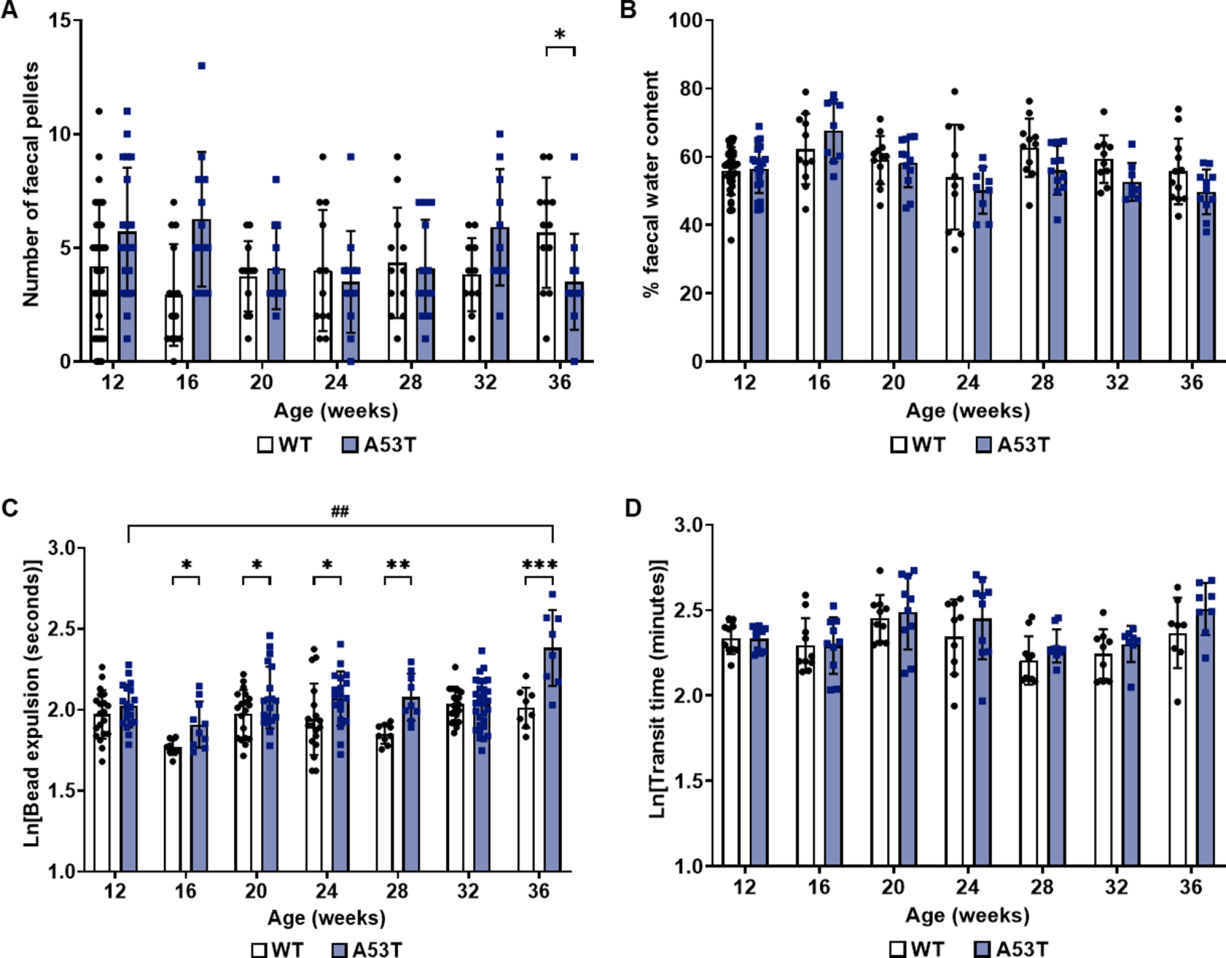
Assessment of GI function in WT and A53T mice. (**A**) No significant difference in FPO was observed between WT and A53T mice, except at 36 weeks of age where A53T mice showed reduced output. (**B**) Pellet water content remained consistent across all timepoints for both genotypes. (**C**) A53T mice exhibited a significant increase in bead expulsion time, starting as early as 16 weeks, indicating impaired colonic motility. This increase persisted at all subsequent timepoints, except at 32 weeks. Additionally, an age-dependent increase in bead expulsion time was observed in A53T mice. (**D**) WGTT showed no significant differences between WT and A53T mice. Bead expulsion and WGTT data were transformed using the natural logarithm to normalise the data and reduce skewness, allowing for more accurate statistical analysis. Data were analysed by Kolmogorov-Smirnov (KS) tests for count data (FPO) and two-way ANOVA with Fisher’s LSD test for other genotype comparisons. Data are presented as mean ± SD, with *n* = 8–30 per group. *p < 0.05, **p < 0.01, ***p < 0.001, ^##^*p* < 0.01 for A53T age-dependent comparison

FPO and faecal water content were used to measure colonic function and motility [9]. Using the 30-minute FPO test, no consistent difference in pellet output was observed between genotypes up until 32 weeks of age. However, at 36 weeks of age, A53T mice showed a significant reduction in pellet output compared to age-matched WT mice (Fig. 2A; *p* < 0.05). Interestingly, faecal water content remained unchanged across all timepoints, including 36 weeks (Fig. 2B).

To further assess colonic motility, we employed the bead expulsion test, which measures large intestine contractility and motility [86]. A53T mice demonstrated slowed colonic motility, as indicated by increased bead expulsion times at 16–28 weeks and 36 weeks compared to WT mice (Fig. 2C; *p* < 0.05). Moreover, there was an age-dependent increase in bead expulsion time in A53T mice, with a significant increase in expulsion time at 36 weeks compared to all other timepoints (*p* < 0.001). Interestingly, WGTT revealed no differences between genotypes at any timepoint (Fig. 2D). Overall, these findings indicate that colonic function deteriorates prior to motor symptom onset and that distal colon function is significantly impacted in the A53T mouse model of PD.

### An age-dependent loss in inhibitory motor neurons is observed in the myenteric plexus of the distal ileum and colon in both WT and A53T mice

**Fig. 3:**
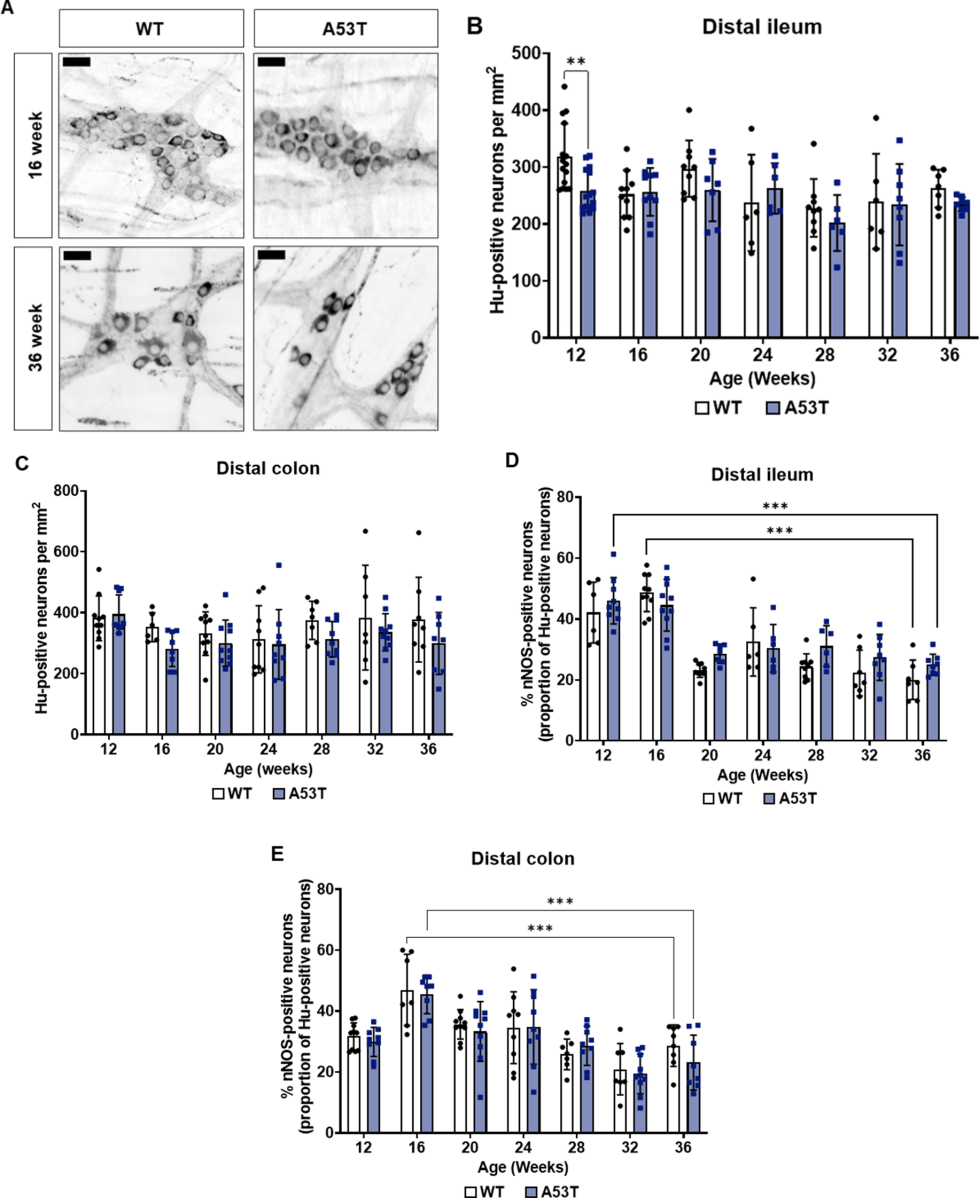
Assessment of enteric neuron number and nNOS-positive neurons in the distal ileum and colon myenteric plexus. (**A**) Representative images of nNOS-positive neurons in the colon of WT and A53T mice at 16 weeks versus 36 weeks. Scalebar = 30 μm. (**B**) An early reduction in the number of Hu-positive neurons per area was observed at 12 weeks in the distal ileum of A53T mice compared to WT mice, with no differences observed at other timepoints. (**C**) No change in the number of Hu-positive neurons per area was found in the distal colon between WT and A53T mice. (**D**) An age-dependent decrease in the proportion of nNOS-positive neurons was observed in the distal ileum for both WT and A53T mice, with no significant genotype effect. (**E**) A similar age-dependent decrease in the proportion of nNOS-positive neurons was seen in the distal colon for both WT and A53T mice, with no significant genotype effect. Data represent mean ± SD; *n* = 6–10 per group. Data were analysed by two-way ANOVA followed by Fisher’s LSD test for genotype comparisons, and one-way ANOVA was performed to assess age-dependent effects, ***p < 0.001

Enteric neuropathy has been linked to GI deficits in animal models of PD [4] as well as human patients [60]. To investigate whether the observed changes in GI function in A53T mice were associated with a progressive loss of enteric neurons, we used the pan-neuronal marker HuC/D and the inhibitory motor neuron marker nNOS to label neurons in the myenteric plexus of the distal ileum and colon. Interestingly, a significant reduction in the number of Hu-positive neurons per area was observed in the ileum of A53T mice at 12 weeks of age when compared to WT mice (*p* < 0.01). However, this reduction was no longer observed at later timepoints (Fig. 3B). Despite the observed deficit in colonic motility identified using the bead expulsion test, no change in enteric neuron number was noted in the distal colon of A53T mice compared to WT mice at any timepoint (Fig. 3C).

The proportion of inhibitory motor neurons in the ileum decreased in an age-dependent manner regardless of genotype (Fig. 3D). Specifically, there was a 28% reduction in WT mice when comparing 16- and 36-week-old mice (*p* < 0.001), and a 21% reduction in A53T mice when comparing 12- and 36-week-old mice (*p* < 0.001). A similar age-dependent reduction in the proportion of nNOS-positive neurons was observed in the colon from 16 weeks of age in both WT and A53T mice, with a maximal reduction of 26% (*p* < 0.001) when comparing 16- and 32-week-old mice for both genotypes (Fig. 3E). However, no significant differences in the proportion of nNOS-positive neurons were observed at any timepoint or region between genotypes. Although the proportion of nNOS-positive neurons significantly reduced with age for both genotypes, this did not coincide with a reduction in total neuron number.

### A53T mice display changes to Hu-positive enteric neuron size in the ileum and colon when compared to WT mice

**Fig. 4:**
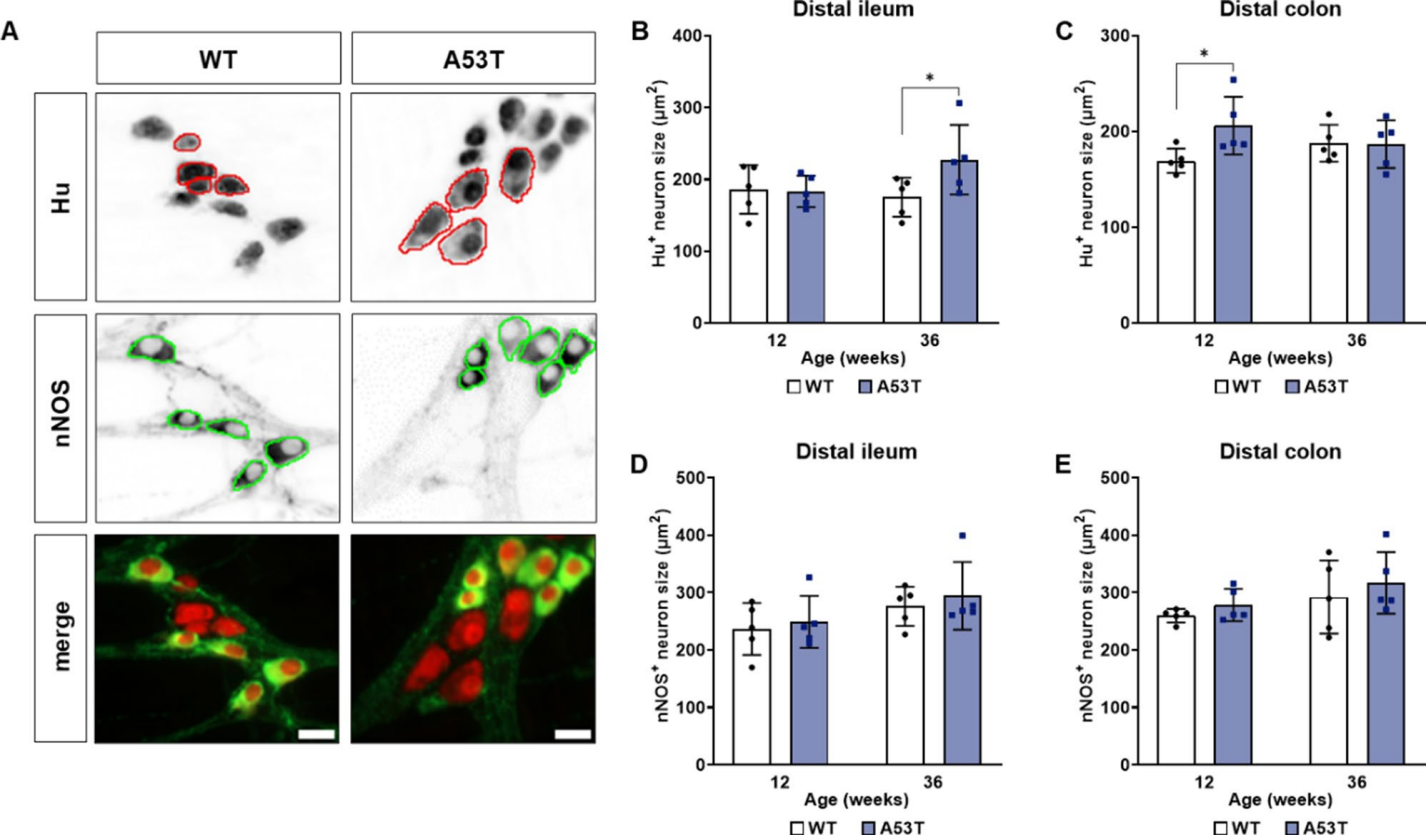
Assessment of neuronal size in the distal ileum and distal colon. (**A**) Representative images of neurons stained for Hu only (outlined with red) and nNOS (outlined with green). Scalebar = 20 μm. (**B**) The average size of Hu-positive enteric neurons in the ileum was larger in A53T mice at 36 weeks compared to WT. (**C**) In contrast, Hu-positive neuron size was larger in A53T mice at 12 weeks when compared to WT mice. (**D**) The average size of nNOS-positive neurons in the ileum was comparable between WT and A53T mice at both 12 and 36 weeks. (**E**) The average size of nNOS-positive neurons in the colon were comparable between WT and A53T mice at both 12 and 36 weeks. Data were analysed by two-way ANOVA followed by Fisher’s LSD test for genotype comparisons. Data represent the mean ± SD, *n* = 5 per group. *p < 0.05

To investigate the potential changes in neuron size in our mouse model, we conducted immunohistochemistry using Hu and nNOS antibodies on ileum and colon samples from WT and A53T mice at 12 and 36 weeks of age. We then analysed the neuron sizes of two populations: neurons that were positive for the pan-neuronal marker HuC/D but not nNOS, and neurons that were positive for both Hu and nNOS (Fig. 4). A significant difference was observed in the former population where A53T mice had larger sizes of Hu-positive neurons compared to WT mice in the ileum at 36 weeks (*p* < 0.01; Fig. 4B) and in the colon at 12 weeks of age (*p* < 0.01; Fig. 4C).

### The level of Hu nuclear translocation was increased in A53T mice

**Fig. 5:**
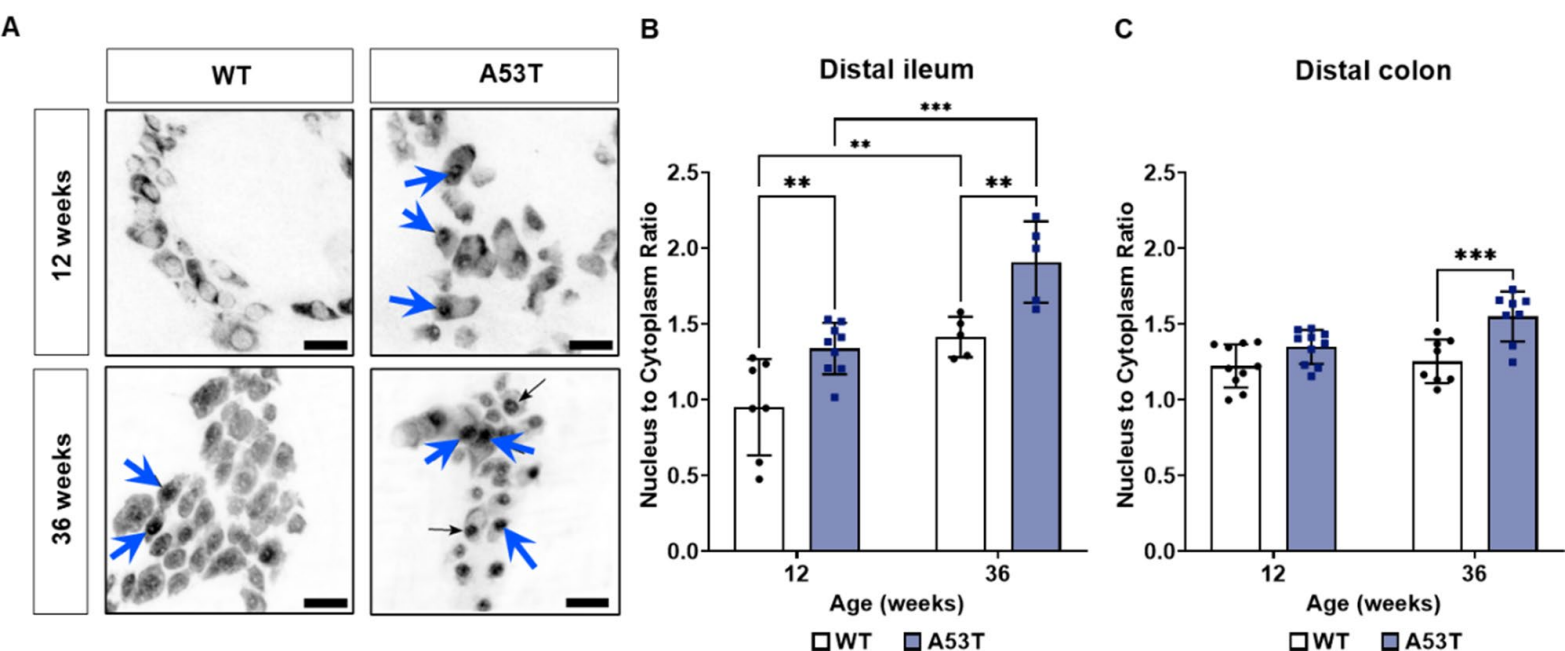
Assessment of Hu-positive nuclear translocation in the distal ileum and distal colon (**A**) Representative photomicrographs depicting nuclear translocation (blue arrows) within the myenteric plexus of the distal ileum of 12-week-old and 36-week-old mice. Scalebar = 30 μm. (**B**) The ratio of Hu-positive translocation from the cytoplasm to the nucleus was higher in enteric neurons from A53T mice at both 12 and 36 weeks in the distal ileum when compared to WT mice. (**C**) In the colon, Hu-positive translocation increased at 36 weeks in A53T mice when compared to WT mice. Data were analysed by two-way ANOVA, Fisher’s LSD test for genotype comparisons. Data represent the mean ± SD (*n* = 5–10 per group). **p < 0.01, ***p < 0.001

Hu translocation from the cytoplasm to the nucleus was assessed as a measure of neuronal stress. Hu translocation from the cytoplasm to the nucleus was assessed as a measure of neuronal stress. Our study revealed significantly higher level of Hu translocation in A53T mice compared to age-matched WT mice at both 12 (*p* = 0.003) and 36 weeks (*p* = 0.003) in the ileum as observed by an increased nucleus to cytoplasm ratio (Fig. 5B). In the colon, there was a significant difference in translocation at 36 weeks (*p* < 0.001), but not at 12 weeks. Our study revealed a significantly higher level of Hu translocation in A53T mice compared to age-matched WT mice at both 12 and 36 weeks in the ileum and at 36 weeks in the colon (Fig. 5C).

### Enteric glial cell number was reduced in young A53T mice

**Fig. 6:**
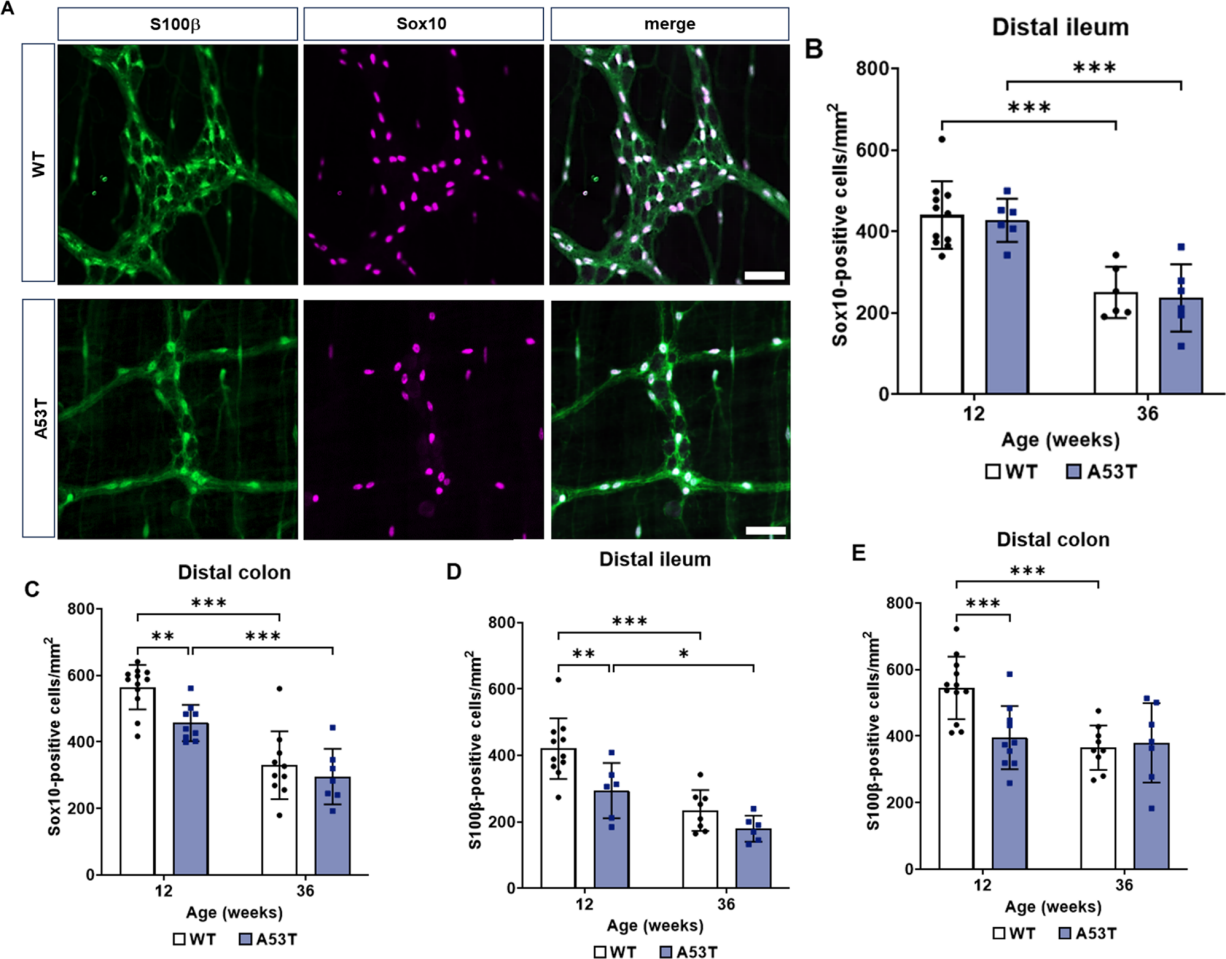
Assessment of enteric glial cell density in WT and A53T mice. (**A**) Representative images of myenteric plexus preparations from the distal ileum of WT and A53T mice stained with the enteric glial markers Sox10 (green) and S100β (magenta). Scalebar = 50 μm. (**B**) There was no genotype-dependent change in the numbers of Sox10-positive glial cells in the distal ileum, however, an age-dependent reduction was observed for both genotypes. (**C**) In the colon, an early reduction in Sox10-positive glial cells was observed in A53T mice at 12 weeks when compared to WT mice, however, this reduction was not maintained at 36 weeks. Similar to the ileum, an age-dependent reduction in Sox10-positive cells was observed for both genotypes. (**D**) An early reduction in the number of s100β-positive glial cells was observed for A53T mice at 12 weeks of age when compared to WT mice. This reduction was not maintained at 36 weeks; however, an age-dependent reduction in s100β-positive cells was observed for both genotypes. (**E**) An early reduction in s100β-positive cells in A53T mice was also observed in the distal colon at 12 weeks when compared to WT mice, however an age-dependent decrease in s100β-positive cells was only observed in WT mice. Data were analysed by two-way ANOVA followed by Fisher’s LSD test for genotype comparisons. Data are shown as mean ± SD, *n* = 8–10 per group, *p < 0.05, **p < 0.01, ***p < 0.001

To determine if changes in glial cell number potentially contribute to the early deficits in GI function observed in A53T mice, we assessed the number of glial cells/mm^2^ in the myenteric plexus of the ileum and colon. Given that different subtypes of myenteric glia express glial markers variably, we used Sox10 and S100β, two of the most widely expressed enteric glial cell markers, for quantification [16] (Fig. 6A). There was no difference in the number of Sox10 immunoreactive glial cells in the distal ileum between WT and A53T mice. However, an age-dependent decrease was noted in both genotypes (Fig. 6B; *p* < 0.001). Interestingly, significantly fewer number of Sox10-positive glial cells were observed at 12 weeks in the colon of A53T mice when compared to WT mice (*p* < 0.01), but this reduction was no longer present by 36 weeks (Fig. 6C). An age-dependent reduction in Sox10-positive cells was observed in the colon for both genotypes (Fig. 6C; *p* < 0.001).

Interestingly, an early reduction in the number of S100β-positive glial cells was observed at 12 weeks in A53T mice compared to WT mice in both the ileum and colon (Fig. 6D and E; *p* < 0.001). An age-dependent decrease in S100β was observed for both genotypes in the ileum (Fig. 6D), and a similar decrease was observed in the colon, but only for WT mice (Fig. 6E).

### Ca2+ transients were increased in young A53T mice

**Fig. 7:**
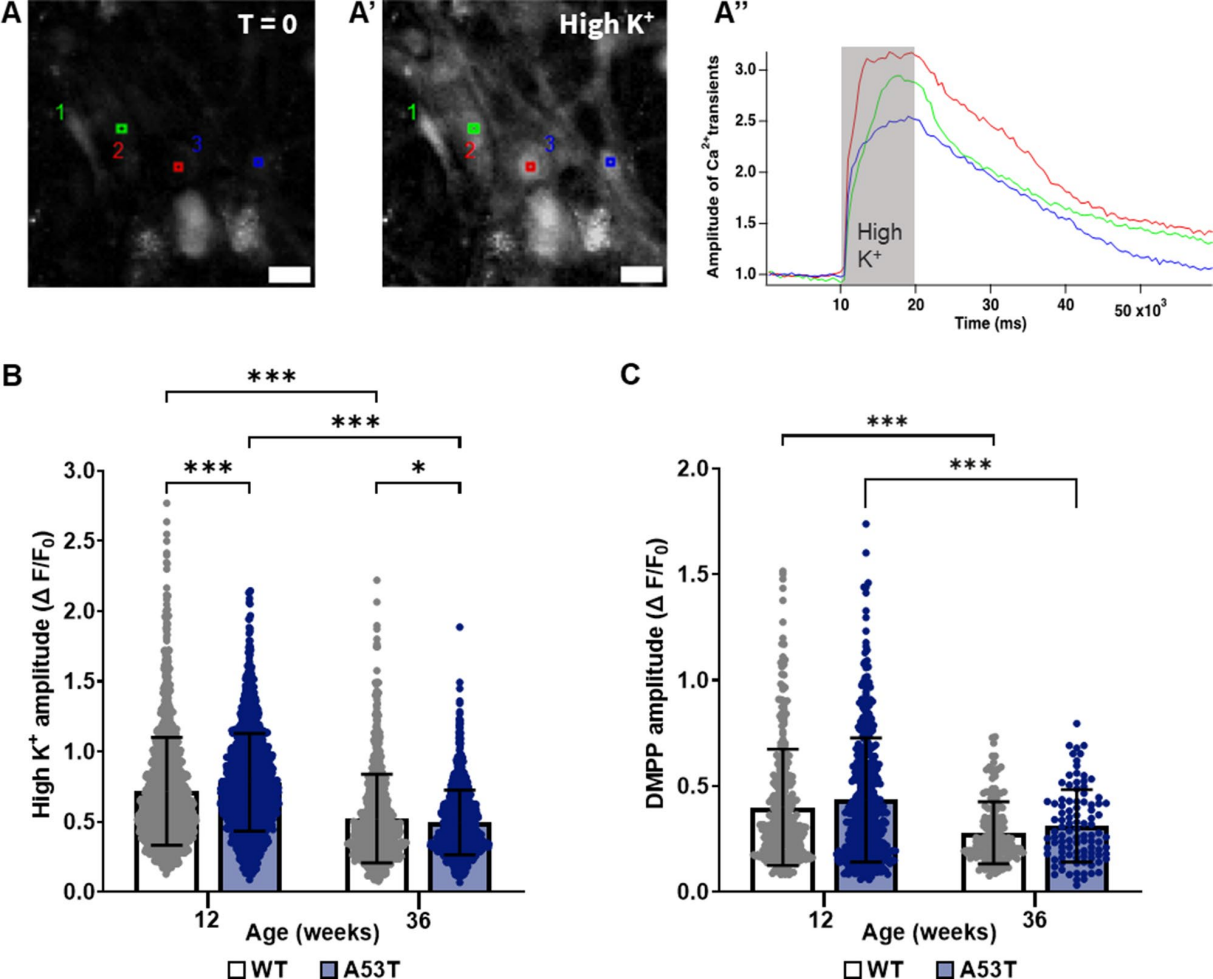
Assessment of Ca^2+^ transients in A53T and WT mice. (A-A”) Representative images of enteric neurons with analysis ROIs at before; t = 0 (**A**), following depolarisation with high K^+^ (A’), and representative color-coded traces corresponding to each ROI (A”). Scalebar = 30 μm. (**B**) The amplitude of Ca^2+^ transients were higher in A53T mice compared to WT mice in response to high K^+^ depolarisation at 12 weeks of age. In contrast, WT mice exhibited higher Ca^2+^ transients at 36 weeks when compared to A53T mice. An age-dependent reduction in Ca^2+^ transients was observed for both genotypes. (**C**) No genotype-dependent change in calcium signalling was observed in response to DMPP, but an age-dependent reduction was evident for both WT and A53T mice. Responses from individual neurons are indicated by dot values, with mean ± SD shown by bar graphs. Data were analysed by two-way ANOVA followed by Fisher’s LSD test for multiple comparisons. Data represent mean ± SD; For high K^+^, *n* = 1588 and 1574 neurons for WT (*n* = 6 mice) and A53T (*n* = 5 mice), respectively, at 12 weeks; *n* = 700 and 1004 neurons for WT (*n* = 10 mice)and A53T (*n* = 10 mice), respectively, at 36 weeks; For DMPP, *n* = 349 and 462 neurons for WT (*n* = 6 mice) and A53T (*n* = 5 mice), respectively, at 12 weeks; *n* = 169 and 99 neurons for WT (*n* = 4 mice) and A53T (*n* = 4 mice), respectively, at 36 weeks, *p < 0.05, ***p < 0.001

Intracellular calcium signalling in enteric neurons of the distal colon were assessed ex vivo using the calcium indicator Fluo4AM to investigate neuronal function (Fig. 7A). Enteric neurons from A53T mice exhibited approximately 10% (significantly) higher Ca^2+^ signalling peak in response to a short high K^+^ depolarisation (10 s) compared to WT mice (Fig. 7B; *p* < 0.001, *n* = 5–6 mice). However, this increased response was reversed with ageing, as WT mice showed a higher Ca^2+^ signalling peak than A53T mice at 36 weeks (*p* < 0.05, *n* = 10 mice). An age-dependent decrease in Ca^2+^ signalling was observed for both genotypes (Fig. 7B; *p* < 0.0001). In contrast to the high K^+^, no genotype-dependent differences were observed in response to DMPP, however, a similar age-dependent reduction in Ca^2+^ signalling was noted for both genotypes (Fig. 7C; *p* < 0.0001).

### Phosphorylated α-Syn is present in the ileum and colon of A53T mice

**Fig. 8:**
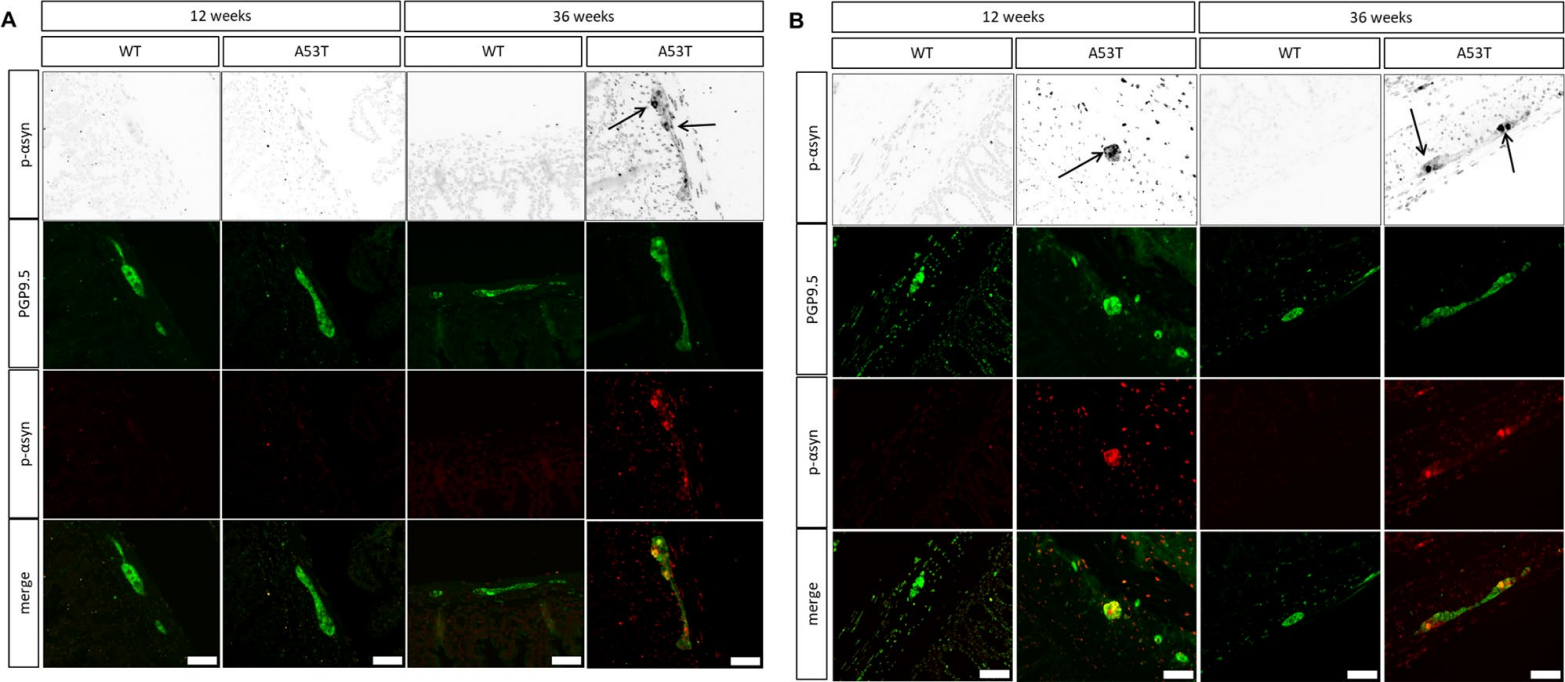
Presence of phosphorylated α-Syn in the myenteric plexus of A53T mice. (A, B) Representative images of myenteric plexus in WT and A53T mice labelled with Protein gene product (PGP) 9.5 and phosphorylated α-Syn at 12 and 36 weeks. At 36 weeks, phosphorylated α-Syn was detected exclusively in the ileum of A53T mice, not in WT mice (**A**). Phosphorylated α-Syn was only detected in the colon of A53T mice but not WT mice at both 12 and 36 weeks (**B**)

The presence of pathological α-Syn in the myenteric plexus was evaluated through immunostaining for phosphorylated α-Syn (Fig. 8). No pathological α-Syn was observed in the ileum of WT or A53T mice at 12 weeks; however, it appeared in the myenteric plexus of A53T mice by 36 weeks (Fig. 8A). In the colon, pathological α-Syn was present in A53T mice at both 12 and 36 weeks (Fig. 8B) yet was absent in WT mice at both time points, preceding the emergence of colonic dysmotility in A53T mice at 16 weeks.

## Discussion

GI dysfunctions in PD were first documented over 200 years ago in James Parkinson’s seminal essay on shaking palsy [82]. Since then, numerous studies have aimed to understand the pathological mechanisms involved in PD-related GI deficits and determine if these symptoms can be used as predictors of disease [14, 18, 33, 34]. Studies using chemically induced and transgenic animal models of PD indicate that GI function is significantly altered as disease-like pathology progresses in the CNS, similar to observations in PD patients [21, 31, 37, 61, 83]. However, the precise role of the ENS in PD-related GI deficits and the timing of their onset remains unclear. Thus, we aimed to determine the timing of GI symptom onset and its temporal relationship with CNS phenotype in the A53T mouse model using functional assays and assessments at both the tissue and cellular levels. It should be noted that the levels of expression of the A53T gene or the levels of A53T protein have not been studied in different regions of the brain and gut. However, the levels of protein deposition are differential [45, 104]. This makes the comparison of timing of symptoms in the mouse complicated.

Similar to previous studies, the ledged beam test showed that A53T mice develop a motor deficit at approximately 36 weeks of age [44, 66, 75, 77, 83], this deficit coincided with a loss of SNpc neurons at 32–40 weeks. Interestingly, colonic motility deficits, as assessed using the bead expulsion test, appeared prior to the onset of motor dysfunction and central neurodegeneration, with A53T mice having slower bead expulsion times from 16 weeks of age when compared to WT. This early deficit in colon function is similar to that observed in another transgenic mouse line, which carries a P1 artificial chromosome with the human A53T mutation in the *SNCA* gene. In this line, mice exhibit colonic motility deficits at 12 weeks of age [57]. In addition, an age-dependent reduction in motility was only observed in A53T mice, with colonic function in WT mice remaining consistent throughout the 24 weeks of testing, indicating that the overexpression of mutant α-Syn may have an early and sustained impact on colorectal function.

To further investigate the early impacts of α-Synuclein overexpression on gastrointestinal function, we expanded our investigation into WGTT. Transgenic A53T mice have been shown to display prolonged WGTT as early as 12 weeks of age, as observed in the G2-3 mutant human A53T mouse line [89] as well as in mouse lines generated by insertion of the entire mutant human *SNCA* gene [57]. However, in the M83 line used in our study, WGTT has only been assessed in older mice (15–22 months) [31, 32, 105]. In contrast to these studies, our results did not show a significant increase in WGTT in A53T mice compared to WT mice suggesting strain-specific differences. However, there was a trend toward increased WGTT in A53T mice at all time points, except at 12 weeks. This trend suggests that subtle alterations in regional gut motility occur in the absence of overt, measurable deficits in whole-gut transit at the specific timepoints assessed. The lack of statistical significance observed in our study may be attributed to the variability inherent in measuring complex motility patterns influenced by multiple factors, including compensatory mechanisms in different gut regions. Additionally, WGTT represents an integrative measure of motility across the entire GI tract, potentially masking region-specific dysfunctions, such as the significant colonic motility deficits detected using the bead expulsion test. Future studies incorporating high-resolution motility tracking or segmental transit assessments may provide greater sensitivity in detecting region-specific dysmotility in this model. When we conducted additional upper GI transit tests with cochineal dye at 12 and 36 weeks to explore this further, the results were comparable between WT and A53T mice (data not shown). It should be noted that whole gut transit and motility can be affected by factors beyond the ENS, such as diet [26, 27], husbandry, the gut microbiome [54, 55, 111] and host genetics [64, 110]. These variables add complexity to the task of accurately assessing the differences attributable to genotype.

The number of faecal pellets produced remained comparable between WT and A53T mice until they reached 36 weeks of age. At this age, A53T mice exhibited a significant reduction in pellet output, coinciding with a pronounced deficit in colonic motility, as measured by the bead expulsion test. While a decrease in colonic motility is often associated with reduced FPO it is important to recognise that the FPO test involves relocating mice to a novel environment, which can induce a stress response. Thus, it is not surprising that the changes observed with the bead expulsion test, which more accurately measures intrinsic reflexes, were not mirrored in the FPO test, which also assesses CNS-driven defecation responses. Since FPO in a fresh cage has a behavioural component, the observed deficit at 36 weeks is likely due to exacerbated intrinsic propulsive activity deficits in the colorectum, consistently detected by the bead expulsion test from 16 weeks of age. Despite the reduction in colon function, our study demonstrated no significant reduction in pellet water content, a common indicator of constipation [3, 7]. This contrasts with previous studies where A53T mice showed reduced pellet frequency as early as 12 weeks [89], and decreased total pellet weight and water content at 24 weeks [57]. Whilst the results of this study are at odds with previously published works, it is important to note that levels of A53T α-Syn expression in different mouse lines have been linked to the age of disease onset and the severity of disease phenotype [62]. Moreover, genomic DNA levels of the A53T transgene have been correlated with severity of non-motor symptoms [109], with differing levels of Genomic DNA among individual mice contributing to the heterogeneity in functional changes. Similarly, variations in the age of motor phenotype onset within the same mouse line used in our study have also been shown [43]. Thus, while A53T mouse models provides a valuable platform for studying PD-related GI dysfunction, it is important to acknowledge the variability in phenotypic expression across different A53T transgenic lines. The presence of heterogeneous phenotypes among genetically similar mice is not uncommon and not specific to A53T transgenic lines, but it does highlight the need for thorough comparative analysis across different transgenic models to better characterise variations.

PD-related GI deficits have been suggested to be a consequence of enteric neuropathy, with evidence of enteric neuronal loss in the gut of PD patients [60, 97]. To determine if the colonic dysfunction observed in our study was associated with enteric neuron loss, we assessed changes in total enteric neuronal populations and inhibitory motor neurons. The total number of enteric neurons was assessed by quantifying immunoreactivity to the pan-neuronal DNA binding protein HuC/D, previously used to assess for neurodegeneration in the ENS [88]. Interestingly, we did not see any significant differences in the total number of myenteric neurons in the ileum or colon when comparing age-matched WT and A53T mice at any timepoint consistent with findings from patient biopsies [6]. Findings from studies using rodent models of PD show a wide range of outcomes, with some reporting an increase, others a decrease, and some finding no change in specific neuronal populations in MPTP [23, 35] and 6-hydroxydopamine (6-OHDA) models [25]. The inconsistency across studies may be attributable to the differing models and stages of the disease.

Inhibitory neurons containing nNOS are essential for the regulation of the inhibitory neurotransmitter nitric oxide from the myenteric plexus [39, 101]. They are more vulnerable to oxidative stress [88] and abnormalities in nNOS expression are known to disrupt colonic peristalsis and transit [74]. In this study, the proportion of nNOS-immunoreactive neurons in A53T mice was comparable to WT at all timepoints observed. Collectively, these findings indicate that the observed deficits in colon motility were most likely not linked to alterations in Hu and nNOS neuron populations. Thus, future studies should incorporate the use of markers that identify other enteric neuron populations, such as excitatory neurons, interneuron populations, and afferent neurons, which will provide further insights into the regulation of colonic function in this mouse model. Interestingly, an age-dependent reduction in the proportion of nNOS-positive neurons was observed for both WT and A53T mice in the ileum and colon. In rats, the proportion of nNOS-positive immunoreactive area in the submucosal border, circular muscle and myenteric plexus was shown to decrease at 31 weeks of age compared to 6 weeks [55]. Therefore, the proportional decrease with age seen in our study is not unexpected, but is unlikely to be due to neuronal loss, as the total number of enteric neurons remained consistent across all timepoints. Instead, it is most likely attributed to changes in nNOS expression levels.

The morphology of enteric neurons in PD models has not been extensively studied. However, in other synucleinopathies like multiple system atrophy, which also presents with Parkinsonian symptoms including GI symptoms, enteric neuronal size has been shown to shrink [80]. And we have previously shown that cell body size of enteric neurons were larger in 32-week-old A53T mice [73]. These alterations in neuron size may reflect underlying neuronal damage or stress [90]. Our results here showed that A53T mice possessed significantly larger Hu-positive neurons in the ileum at 36 weeks and in the colon at 12 weeks. Similar findings of increased soma size in the colon were observed in 32-week-old A53T mice [72]. Additionally, in neuroblastoma cell lines, neuronal cells expressing mutant A53T α-Syn or W437X Pink1 showed significant increases in mitochondrial size, which could contribute to alterations in neuronal profiles [68]. This transient hypertrophic morphology may result from the accumulation of aggregated α-Syn, warranting further investigation through immunohistochemical analysis.

Nuclear translocation of Hu protein has been observed in enteric neurons under conditions associated with neuronal toxicity and stress, such as ischemia and chemotherapy treatments [17, 30, 88]. When Hu proteins are localised in the cytoplasm, they regulate mRNA translation and prevent the destabilising effects of certain proteins. However, their presence in the nucleus can indicate imminent mRNA degradation and cellular toxicity [52]. Our study revealed significantly higher levels of Hu translocation in the ileum at 12 and 36 weeks, and in the colon at 36 weeks in A53T mice. These findings contrast with previous studies [31, 72], where no difference in Hu translocation was found. This discrepancy accentuates the heterogeneity within the mouse line used in our study, as well as the potential genetic drift that may have occurred over time within this particular line. However, the presence of this neuronal stress indicator at 36 weeks coincides with significantly increased colonic dysmotility as well as decreased pellet output implicating neuropathy in GI dysfunction. These findings underscore the dynamic nature of neuronal adaptations to PD pathology. Early compensatory hypertrophy may help maintain function against increasing α-Syn burden, but as stress accumulates, these mechanisms may fail, leading to a decline in functional deficits.

Following these observations, we further investigated whether colonic motility deficits and neuronal stress were accompanied by changes in enteric neuronal activity. While calcium imaging has been used to investigate the functional differences in CNS neurons of PD animal models [30, 100], few studies have assessed calcium signalling in enteric neurons. Our findings revealed that the calcium response to cell depolarisation by high K^+^ solution was significantly different between control vs. A53T mice. In the CNS, α-Syn has been shown to cause Ca^2+^ influx likely through its direct interaction with N-type Ca^2+^ channels, which affects voltage-dependent Ca^2+^ channels [1]. α-Syn interacts with membranes to affect Ca^2+^ signalling in a structure-specific manner. Oligomeric β-sheet-rich α-synuclein species lead to Ca^2+^ dysregulation and Ca^2+^-dependent cell death [5]. These oligomeric species can spontaneously form calcium-permeable pores that are partially voltage-dependent, increasing membrane transduction and cytosolic Ca^2+^ uptake [103]. These pores can form even at resting membrane potentials, contributing to neurotoxicity by disrupting cellular ion homeostasis and inducing cellular stress [67]. Thus, it is plausible that overexpression of the human A53T variant alters enteric neuron function early on in disease progression. Due to technical difficulties, the current study is lacking post-hoc identification of neurons after calcium imaging. Whether these differences in responses are localised to specific regions of the gut remains to be investigated further; previously, it has been shown that there were no significant changes in the calcium activity of submucous neurons isolated from the duodenum of PD patients [29]. Nonetheless, these findings highlight the importance of assessing functional changes in enteric neurons such as whole-cell patch-clamp electrophysiology [79], rather than focusing solely on gross neuronal loss or changes in neuron size.

Enteric glial cells also have key roles in gastrointestinal function. In addition to providing structural and metabolic support to enteric neurons, they are also important for neuronal function [49], contributing to the coordination of muscle contractions and relaxation in the GI tract [48, 50]. In addition to enteric neurons, enteric glial cells also fulfil diverse roles for GI function by providing neuronal support, maintaining mucosal integrity, and contributing to neuroprotection, neurogenesis, and synaptic transmission [24, 28, 42, 87, 102]. However, little research has been undertaken to investigate the involvement of enteric glial cells in PD-related GI dysfunction, especially in the A53T mouse model. In alignment with prior human studies linking constipation to the degeneration of enteric glial cells in patients with intractable constipation [10, 11], our study found decreased populations of intra-ganglionic myenteric glial cells in both the ileum and colon in young A53T mice, as well as in both genotypes of mice with age. This population of enteric glial cell is specifically known for modulating myenteric neuron activity, regulating oxidative stress, providing trophic support, regulating neuroinflammation, gliogenesis, and neurogenesis as well as replenishment of mucosal glia [76]. These enteric glial cells are poised to differentiate into neurons under certain conditions and act as the neural stem cells of the gut [51]. The early reduction in colon glial cells at 12 weeks was consistent for both S100β and Sox10 localisation, providing further evidence of early changes at the level of the ENS. Depletion of enteric glia and inhibition of glial communication has previously been shown to influence gut motility [70, 71]. There is evidence to support the age-related decline of enteric glial cell populations [84, 91, 99], In addition, alterations in ATP-mediated glial communication has been shown to reduce with age, which correlate with the age related decreases in gut motility [70]. Our findings in both WT and A53T mice support the documented decline in enteric glial cell populations associated with ageing. Notably, we also observed a concurrent decline in GI function. However, further investigation is required to comprehensively elucidate the mechanisms that underlie the interplay between the two. Building on these observations, the presence of phosphorylated α-Syn in the myenteric plexus of the ileum and colon offers further evidence of progressive neuropathy in the gut, potentially driving functional deficits. Specifically, α-Syn was consistently detected in the colonic myenteric plexus by 12 weeks, which aligns with the emergence of colonic motility deficits by 16 weeks. This finding is supported by our calcium imaging studies, which revealed altered Ca^2+^ signalling responses in A53T mice, resembling disruptions observed in central nervous system neurons. The functional effects of α-Syn on enteric neurons likely mirror its role in CNS pathophysiology, where its interaction with Ca^2+^ channels and membranes lead to dysregulated Ca^2+^ signalling and subsequent neurotoxicity. Moreover, the early depletion of intra-ganglionic myenteric glial cells observed in the colon and ileum at 12 weeks—coupled with age-related declines—underscores the critical role of enteric glial cells in maintaining neuronal health and motility. These collective insights highlight the need for further exploration into region-specific enteric neuron and glial changes, as well as functional studies beyond structural assessments, to fully understand how α-Syn pathology and enteric glial alterations impact gut motility deficits in Parkinsonian models.

### Limitations

While this study provides novel insights into GI dysfunction and alterations to ENS in the A53T mouse model of PD, several limitations should be acknowledged. First, the absence of post-hoc identification of neurons following calcium imaging represents a methodological limitation. Without definitive confirmation of neuronal subtypes, it remains unclear whether the observed differences in calcium transients are specific to particular neuronal populations. Future studies employing immunohistochemical co-labelling or genetically encoded calcium indicators in distinct neuronal subsets should be conducted. Second, our study focused on colonic dysfunction, with limited assessment of compensatory mechanisms in non-colonic regions. It is possible that the absence of whole-gut transit differences reflects adaptive responses in the upper gastrointestinal tract, however additional studies assessing gastric emptying, small intestinal motility, and vagal-brainstem interactions could provide a more comprehensive understanding of GI dysfunction in this model. Third, the extent to which strain-specific effects or transgene-level variability influence phenotype expression remains an open question. Differences in disease onset and severity have been noted between transgenic models of α-synuclein overexpression, and even within the same model, individual variability exists. The inclusion of multiple transgenic lines and a systematic assessment of α-synuclein expression levels in different gut regions could provide greater clarity on these factors. Further to this, while our findings demonstrate a link between α-synuclein pathology, ENS dysfunction, and GI motility deficits, additional mechanistic studies are needed to elucidate the underlying cellular and molecular pathways driving these changes. Investigating the roles of key signalling pathways known to be implicated in neuroinflammation, oxidative stress, and synaptic dysfunction could provide valuable insights into disease mechanisms. Experimental approaches such as pharmacological inhibition of α-synuclein aggregation, targeted genetic manipulations, or in vitro ENS cultures could help delineate causative relationships and identify potential therapeutic targets for PD-related GI dysfunction. Lastly, the exclusion of human validation studies limits the translational relevance of our findings. While the A53T mouse model recapitulates key aspects of PD-related enteric pathology, direct comparisons with human tissue from PD patients, including assessments of enteric neuron morphology, calcium signalling, and glial changes would strengthen the clinical applicability of our results. Despite these limitations, our findings contribute valuable insights into the early ENS changes preceding motor deficits in PD, highlighting the need for further mechanistic and translational research in this area.

## Conclusion

In conclusion, our study in the A53T PD mouse model revealed colonic motility deficits preceding motor symptoms and nigral cell loss, accompanied by signs of neuronal stress, altered calcium signalling, and reduced enteric glial cells in the absence of overt neuronal loss. The morphological changes in enteric neurons further emphasise complex neuronal adaptations occurring with disease progression. Importantly, these findings diverged from some previous studies, highlighting the heterogeneity within this model. Regardless, these have important implications for understanding GI dysfunction in PD and its potential as an early disease marker. The early emergence of ENS dysfunction in PD raises the possibility of utilizing GI biomarkers for early diagnosis and intervention. Future human studies should explore whether non-invasive tests, such as colonic transit measurements, fecal α-synuclein detection, or enteric neuronal imaging, could serve as early indicators of PD. Additionally, therapeutic strategies targeting ENS pathology, such as neuroprotective agents, microbiome modulation, or gut-targeted α-synuclein inhibitors, may offer novel approaches to combatting GI dysfunction and potentially delaying disease progression. Overall, our work provides evidence for some degree of enteric neuropathy in PD, with multifaceted changes at the level of the ENS potentially driving GI complications. Moreover, our study provides a broader perspective on the model and new insights into the timing and nature of ENS involvement in PD.

## Data Availability

No datasets were generated or analysed during the current study.
